# HKPocket: human kinase pocket database for drug design

**DOI:** 10.1186/s12859-019-3254-y

**Published:** 2019-11-29

**Authors:** Huiwen Wang, Jiadi Qiu, Haoquan Liu, Ying Xu, Ya Jia, Yunjie Zhao

**Affiliations:** Department of Physics, Central China Normal University, Wuhan, 430079 China

**Keywords:** Pocket database, Human kinase proteins, Drug discovery, Side effects

## Abstract

**Background:**

The kinase pocket structural information is important for drug discovery targeting cancer or other diseases. Although some kinase sequence, structure or drug databases have been developed, the databases cannot be directly used in the kinase drug study. Therefore, a comprehensive database of human kinase protein pockets is urgently needed to be developed.

**Results:**

Here, we have developed HKPocket, a comprehensive Human Kinase Pocket database. This database provides sequence, structure, hydrophilic-hydrophobic, critical interactions, and druggability information including 1717 pockets from 255 kinases. We further divided these pockets into 91 pocket clusters using structural and position features in each kinase group. The pocket structural information would be useful for preliminary drug screening. Then, the potential drugs can be further selected and optimized by analyzing the sequence conservation, critical interactions, and hydrophobicity of identified drug pockets. HKPocket also provides online visualization and pse files of all identified pockets.

**Conclusion:**

The HKPocket database would be helpful for drug screening and optimization. Besides, drugs targeting the non-catalytic pockets would cause fewer side effects. HKPocket is available at http://zhaoserver.com.cn/HKPocket/HKPocket.html.

## Background

Kinase proteins are considered as one of the most attractive drug targets for drug discovery targeting cancer, chronic neurodegenerative or other diseases [1–4]. Previous studies have highlighted two major strategies targeting kinases: ATP-binding inhibitors (type I and II) and non-ATP inhibitors (type III and IV) [3, 5]. Currently, most developed drugs are ATP-competitive inhibitors [6, 7]. Andrea et al. performed a systematic analysis of catalytic ATP-binding pockets. Their results showed that ATP-binding pockets are highly conserved [8]. Therefore, the ATP-competitive drugs may inhibit most of the kinase proteins and cause side effects, such as hypertension, hand-foot skin reaction and acute renal failure [9–11]. Type III and type IV inhibitors are usually very selective and have fewer side effects because their targeted binding sites are usually unique to a particular kinase [3, 5, 12]. Thus, there is an urgent need to develop new drugs targeting non-catalytic pockets to reduce side effects.

Computer-aided drug design is widely used in drug development to shorten the time and reduce the cost of experiments [13–22]. There are several existing kinase databases with sequence, structure or drug information. For example, (1) kinase protein databases (the Kinase.com, the Protein Kinase Resource, the Target Informatics Platform and the KinG database) explore the genomics, evolution and function of protein kinases [23–26]; (2) experimental information databases (the Kinase Validation Set, the KINOMEscan data, the PhosphoBase, the KinMutBase, and the Kinase Pathway Database) contain compound bioactivity, phosphorylation and mutation experimental data [27–32]; (3) kinase catalytic pocket databases (the Kinase Knowledgebase and the Kinase-Ligand Interaction Fingerprints and Structure database) studied the structural and sequence features of ATP-binding and closely nearby pockets [33–35]. However, most of the drugs in these databases are ATP-competitive leading to many side effects. In addition, the available kinase information cannot be directly used in the kinase drug study. The well-analyzed kinase structures are still limited. Thus, a comprehensive and updated human kinase pocket database is urgently needed especially for inhibitors targeting non-catalytic pockets with fewer side effects.

Recently, the kinase family is very well covered by tertiary structures, making it possible to perform a systematic analysis of potential selective binding pockets. Here, we performed a systematic analysis of binding pockets from 255 available human kinase structures to provide potential selective binding pockets and developed HKPocket database with sequence, structure, hydrophilic-hydrophobic and druggability information for kinase drug design.

## Construction and content

### HKPocket database construction

**Fig. 1:**
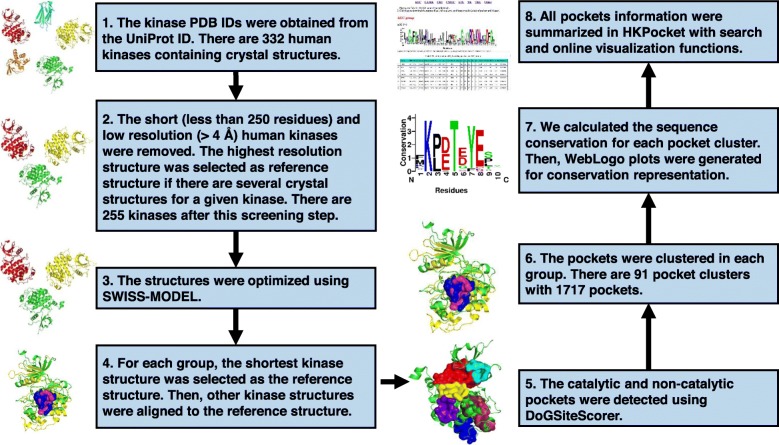
The workflow of the HKPocket database construction

The whole human kinome contains a total of 518 kinases with 478 typical kinases and 40 atypical kinases. The 478 typical kinases were divided into nine groups (AGC: 63, CAMK: 74, CK1: 12, CMGC: 61, RGC: 5, STE: 47, TK: 90, TKL: 43, Other: 83) [36]. A workflow of constructing the HKPocket database is shown in Fig. 1.

**Fig. 2:**
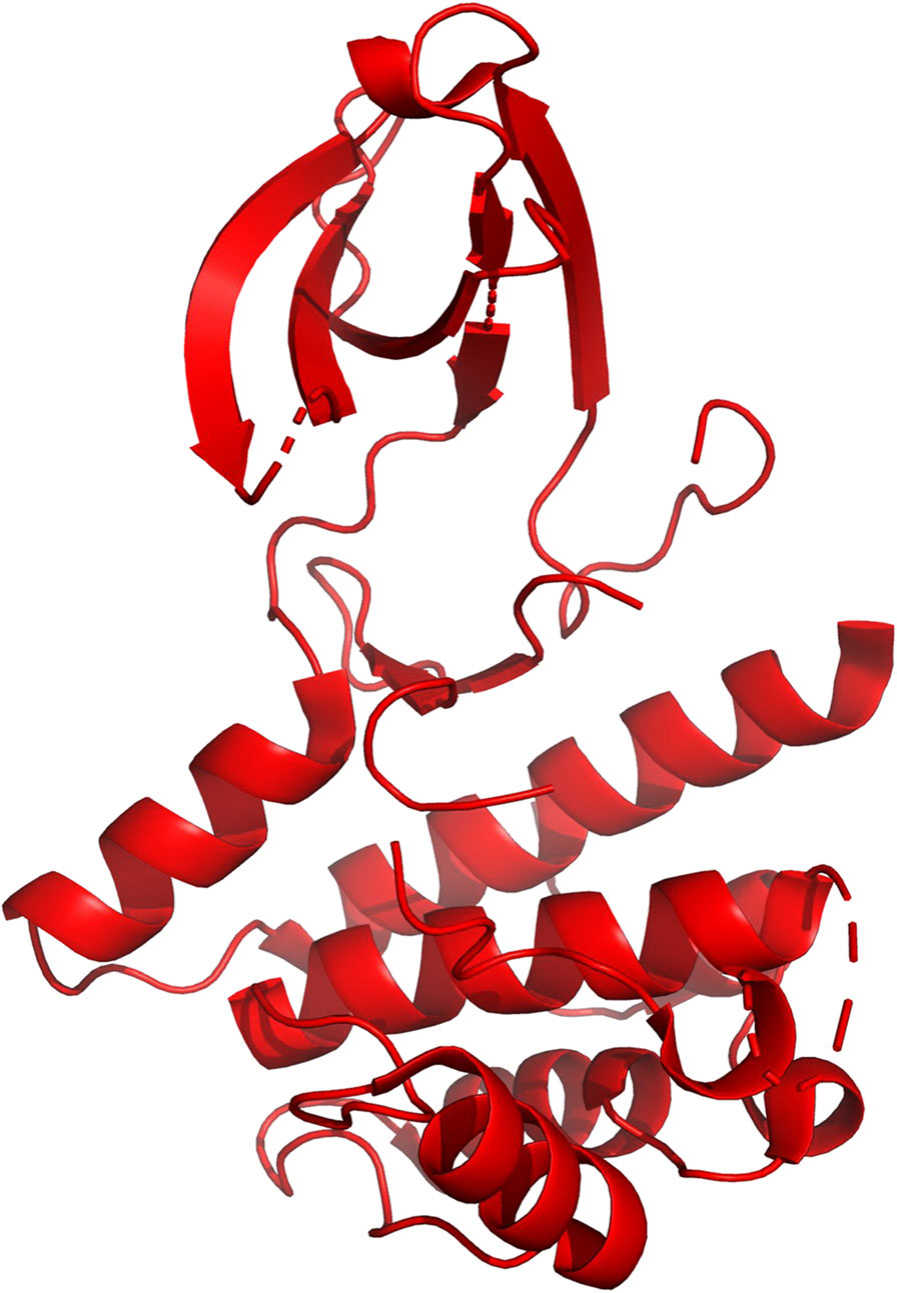
The structure of NEK2 protein kinase. NEK2 (PDB ID: 6H0O) is an incomplete protein with only 219 residues. Therefore, NEK2 does not contain Cα-helix and many loop residues. It is very difficult to identify a pocket using this incomplete structure

**Fig. 3:**
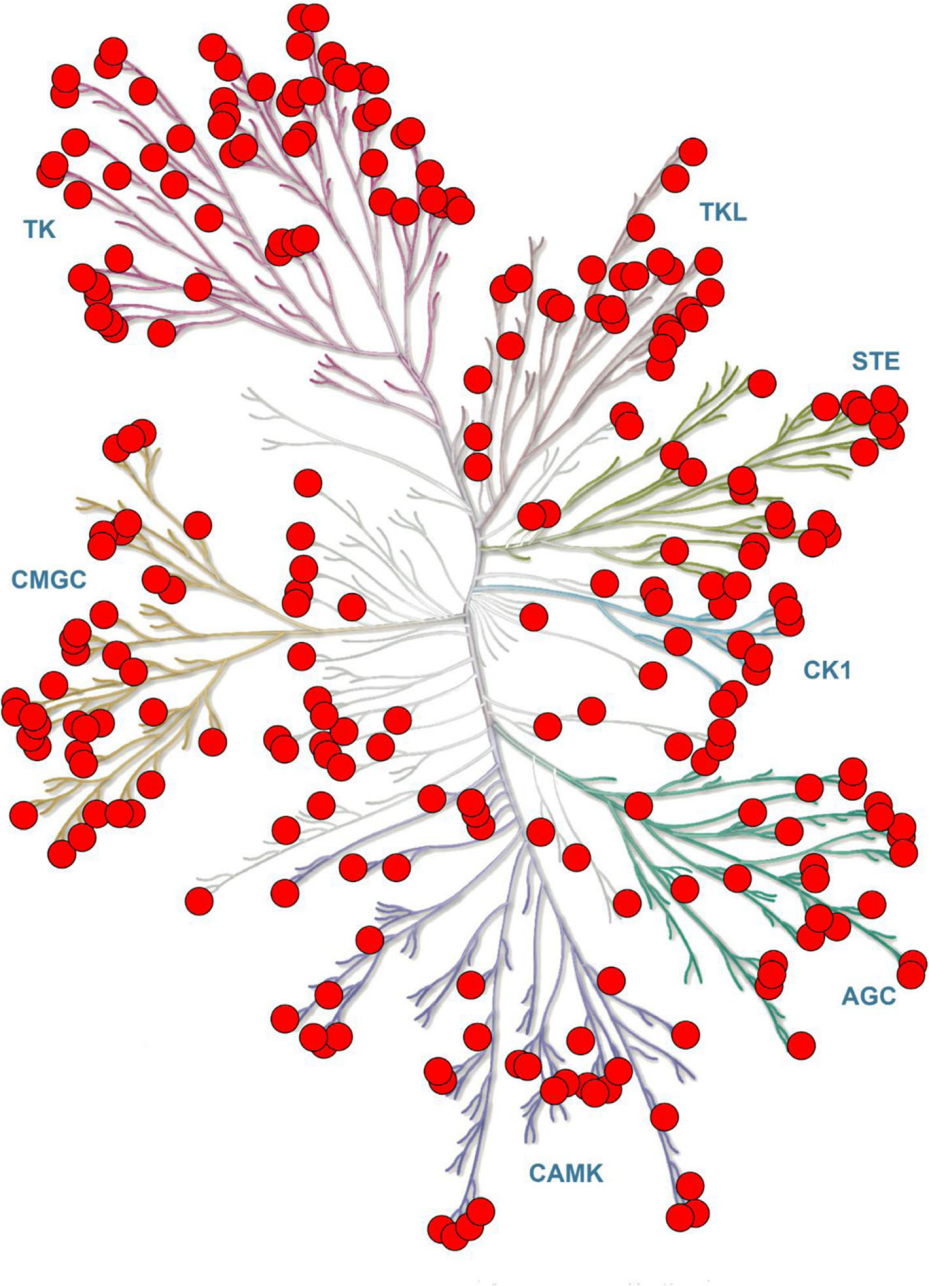
The distribution of 255 human kinases. There is the distribution of 255 human kinases (AGC: 27, CAMK: 36, CK1: 10, CMGC: 37, RGC: 0, STE: 25, TK: 60, TKL: 26, Other: 34) in HKPocket database on human kinome tree. The red dots represent each kinase structure

1. We extracted structures from the PDB (Protein Data Bank) database [37] based on the human kinase UniProt ID [38]. There are 313 human kinase structures (AGC: 41, CAMK: 43, CK1: 10, CMGC: 37, RGC: 0, STE: 31, TK: 73, TKL: 34, Other: 44).
2. We obtained the kinase protein structures by keeping high-resolution structures (resolution < 4 Å) and removing the short proteins with the length fewer than 250 residues. We considered the length cut off based on the following considerations: (i) 90% of kinase proteins are larger than 250 residues [39, 40]. (ii) it is very difficult to determine the pocket information using short kinase proteins with many missing residues. For example, pocket information cannot be extracted from short NEK2 protein kinase (PDB ID: 6H0O) without Cα-helix and other residues (Fig. 2) [41]. The structure with the highest resolution was selected if there are several structures for the same kinase. There are remaining 255 kinase proteins showed in Fig. 3 (AGC: 27, CAMK: 36, CK1: 10, CMGC: 37, RGC: 0, STE: 25, TK: 60, TKL: 26, Other: 34).
3. All the 255 kinase proteins were optimized to fill in the missing atoms using the template-based structure modeling tool SWISS-MODEL [42].
4. The protein with the shortest sequence length was selected as the reference structure in each group. The remaining kinase structures were aligned to the reference structure in the corresponding group.
5. All kinase pockets were detected by DoGSiteScorer which uses a Gaussian filter to detect drug pockets and define drug pocket features [43, 44]. There are 6347 identified pockets from 255 available human kinase structures.
6. The pse files were generated by PyMOL (www.pymol.org) for kinases and their pockets visualization. The 91 cluster pse files (AGC: 11, CAMK: 9, CK1: 13, CMGC: 10, STE: 12, TK: 8, TKL: 14, Other: 14) contain 1717 detected pockets.
7. The multi-sequence alignment of kinase protein sequences in each group was performed. The sequences of pockets were extracted from the aligned kinase protein sequences. The sequence conservation of pocket was analyzed and generated by WebLogo [45]. The overall height of the sequence symbol indicates the sequence conservation at the particular position.
8. HKPocket database is developed in classical MVC (Model-View-Controller) architecture. The model layer is the database containing sequence, structure, and other pocket information. The View layer is the online visualization application implemented by JSmol. The Controller layer provides search function access to the pocket data designed using REST API.

The HKPocket database will be updated annually and provides sequence, structure, and other information, such as volumes, depths, surface, hydrophilic-hydrophobic, and drug score. All the information can be downloaded from the HKPocket database website. In addition, the HKPocket database provides an online visualization module. Users can scale and rotate the structures by cartoon or spacefill representations.

### Content

#### The differences between HKPocket and existing databases

HKPocket database is a comprehensive human kinase pocket database for drug study against kinase-related diseases. The following differences distinguish the HKPocket database from the existing kinase-related databases (Table 1).

1. The human kinase protein databases

**Table 1:** The differences distinguish HKPocket database from the existing kinase-related databases

Types of Database	Databases	Links	Kinase Protein			Experiment				Pocket Information	
			Sequences	Structures	Evolutionary trees	Molecules	Affinities	Phosphorylation sites	Mutation sites	catalytic	Non- catalytic
Human kinase protein databases	Kinase.com	http://www.kinase.com/	✓		✓						
	Protein Kinase Resource	http://www0.nih.go.jp/mirror/Kinases/pk_home.html	✓	✓							
	Target Informatics Platform (TIP)	http://www.eidogen.com/tcc.php		✓							
	KinG	http://king.mbu.iisc.ernet.in/	✓								
Human kinase experiment databases	Kinase Validation Set	https://www.eidogen.com/kinasednld.php				✓	✓				
	KINOMEscan data	http://lincs.hms.harvard.edu/kinomescan/				✓					
	PhosphoBase	http://www.cbs.dtu.dk/databases/PhosphoBase/pbase2/						✓			
	KinMutBase	http://structure.bmc.lu.se/idbase/KinMutBase/							✓		
	Kinase Pathway Database	http://kinasedb.ontology.ims.u-tokyo.ac.jp/	✓				✓				
Human kinase pocket databases	Kinase Knowledgebase (KKB)	http://www.eidogen.com/kinasekb.php					✓			✓	
	Kinase-Ligand Interaction Fingerprints and Structure database (KLIFS)	http://www.vu-compmedchem.nl/klifs				✓	✓			✓	
	HKPocket	http://zhaoserver.com.cn/HKPocket/HKPocket.html	✓	✓	✓					✓	✓

The Kinase.com provides the sequences and evolutionary trees of 15 kinomes, such as human kinome, mouse kinome, and drosophila kinome [36, 46, 47]. The Protein Kinase Resource includes aligned sequences of 390 eukaryotic protein kinases and a description of 50 protein kinase structures [23]. The Target Informatics Platform (TIP) provides more than 195,000 high-resolution protein structures, covering every major drug target family including proteases, kinases, nuclear receptors, phosphatases, phosphodiesterases, and GPCRs [24]. The KinG database is a comprehensive collection of Ser/Thr/Tyr specific kinases and their similar sequences and provides the sequences, functional domain assignments of kinases [25, 26]. These databases simply provide the sequence, structure and evolutionary information but cannot be directly used in the kinase drug study.

2. The human kinase experiment databases

The Kinase Validation Set contains over 3880 molecule structures and corresponding pIC50 data across three kinase targets (ABL1, SRC, and AURKA) [27]. The KINOMEscan data is a table of all small molecules in the HMS LINCS collection that profiled by KINOMEscan, including links to the raw binding data [48]. The PhosphoBase is a eukaryotic phosphorylation site database [28, 29]. The KinMutBase contains 251 mutations representing 621 patients in protein kinase domains [30, 31]. The Kinase Pathway Database provides functional conservation information, protein-gene/protein/compound interactions in existing databases and papers [32]. These databases provide the phosphorylation, mutation, and binding affinity data but without pocket structural information.

3. The human kinase catalytic pocket databases

The Kinase Knowledgebase (KKB) is a database of kinase structure-activity and chemical synthesis data. This database contains all crystallized catalytic domain structures [35]. The Kinase-Ligand Interaction Fingerprints and Structure database (KLIFS) contains kinase-ligand interaction information, ligand and catalytic pocket structures of kinase proteins [34]. Current databases focus on catalytic pockets (ATP-binding pockets or the pockets closely located at ATP-binding pockets). However, the information on ATP pockets is very limited to drug design.

To bridge this gap, we performed a large-scale analysis of 255 available human kinase structures by systematic pocket detection and comparison. HKPocket contains 1717 identified pockets which 85% are non-ATP pockets. A clustering of non-ATP pockets provides a framework to decipher pockets for further study. The major difference between HKPocket and previous work is that we have performed systematic pocket detection, comparison, annotation and visualization of non-ATP pockets.

#### The features of HKPocket database

We have developed a human kinase pocket database for kinase drug design study. Currently, it contains 1717 pockets from 255 kinases.

1. HKPocket database provides the tertiary structures and the structural topology information (volume, surface, and depth) of 91 pocket clusters. In addition, we also provide other quantitative information such as enclosure, ratios between ellipsoid main axes. Most drug discovery development approaches are based on the lock and key model [49, 50]. The pocket topology information would be useful for preliminary drug screening. For example, Volkamer et al. [8] studied the conservation of ATP-competitive pocket in the human kinome by analyzing the volume, and depth of the ATP-competitive pocket. Therefore, the specificity drug pocket study will promote the development of specific drugs to reduce drug side effects.
2. Second, HKPocket provides sequence conservation analysis, the number of metals and specific elements (carbon, nitrogen, sulfur, oxygen and other atoms). The sequence conservation analysis results of detected pockets are shown in WebLogo format. The overall height of the sequence indicates the frequency and conservation at the corresponding position. The pocket sequences and atomic level information would play important roles for further drug screening.
3. HKPocket also provides interaction information of pockets containing hydrophobic interactions as well as the ratio of apolar, polar, positive, negative amino acids and hydrophobicity. These detail interactions would be helpful for drug optimization, especially for side chain or group optimization.
4. Moreover, the drug scores were calculated using a Support Vector Machine (SVM) model [51, 52]. The drug score represents the druggability of pocket ranging from 0 to1 which the higher score indicating a more druggable pocket.
5. HKPocket provides an online visualization module. Users can scale or rotate the pocket tertiary structures by cartoon or spacefill representations. The key residues can be labeled and highlighted in different colors.

## Utility and discussion

HKPocket provides a user-friendly online server. The server contains seven modules: Home, Search, Visualization, Download, Links, Tutorial, and Contacts. The detail information for each module is as follows.

### Home module

**Fig. 4:**
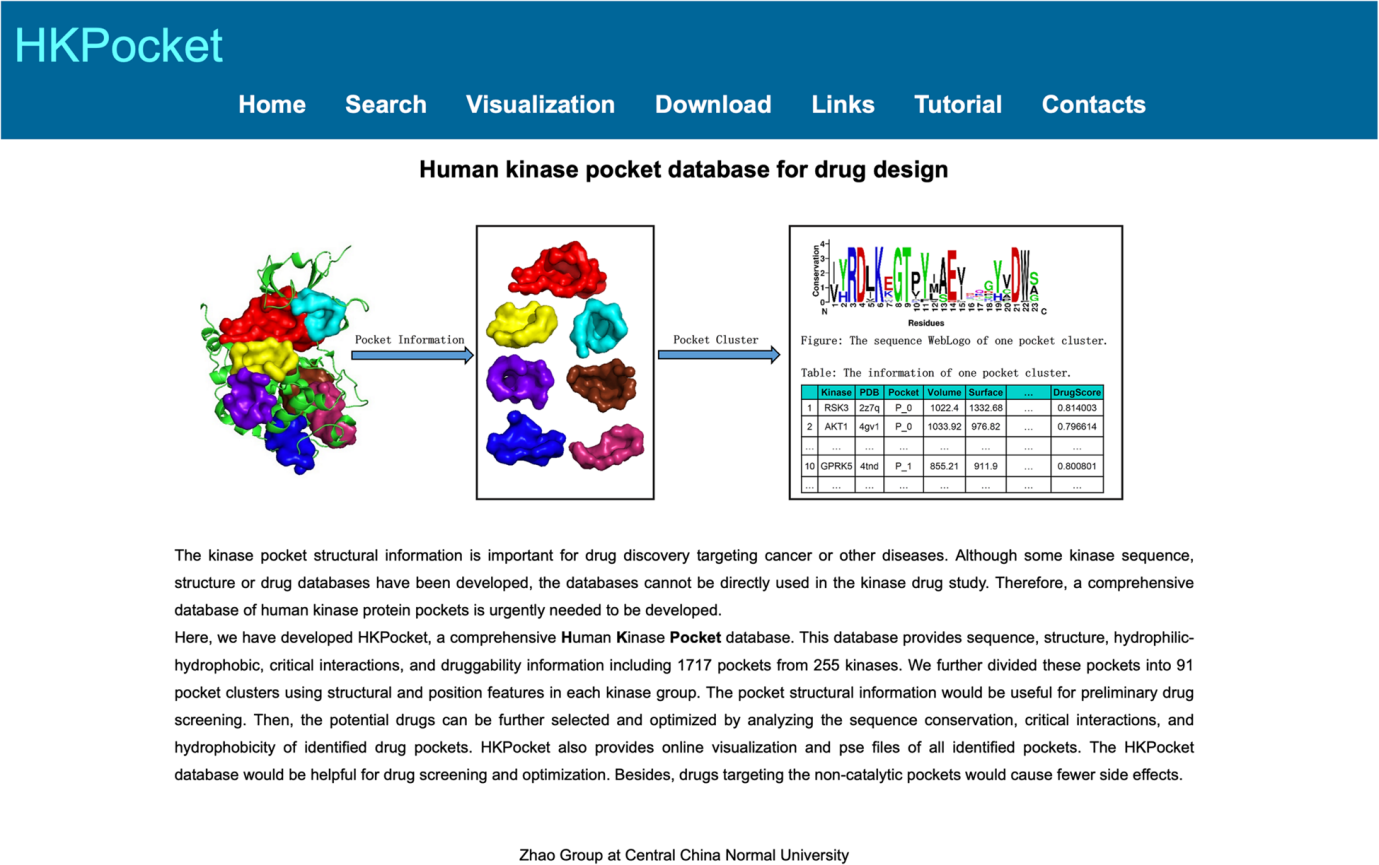
An example of the Home module. The Home module provides an introduction of the HKPocket database. It also provides navigation to other modules of the database

The HKPocket Home module (Fig. 4) provides an introduction to the HKPocket database. It also provides navigation to other HKPocket modules.

### Search module

**Fig. 5:**
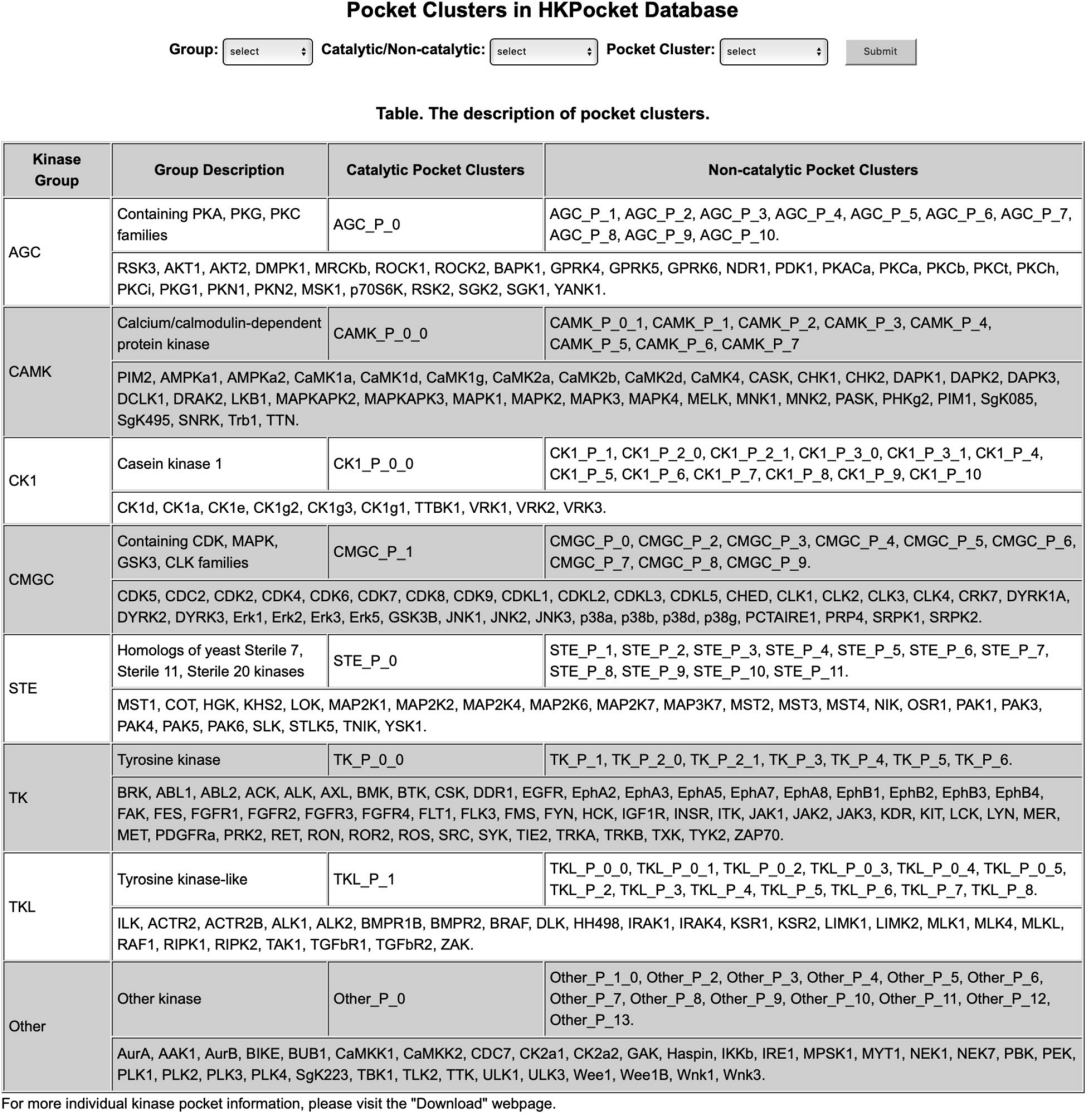
An example of the Search module. The Search module consists of two parts: one pulldown search box and a summary table of pockets. (1) In the pulldown search box, users can select the pocket cluster by group, catalytic/non-catalytic, and pocket cluster information. (2) The summary table contains the names of 91 pocket clusters

**Fig. 6:**
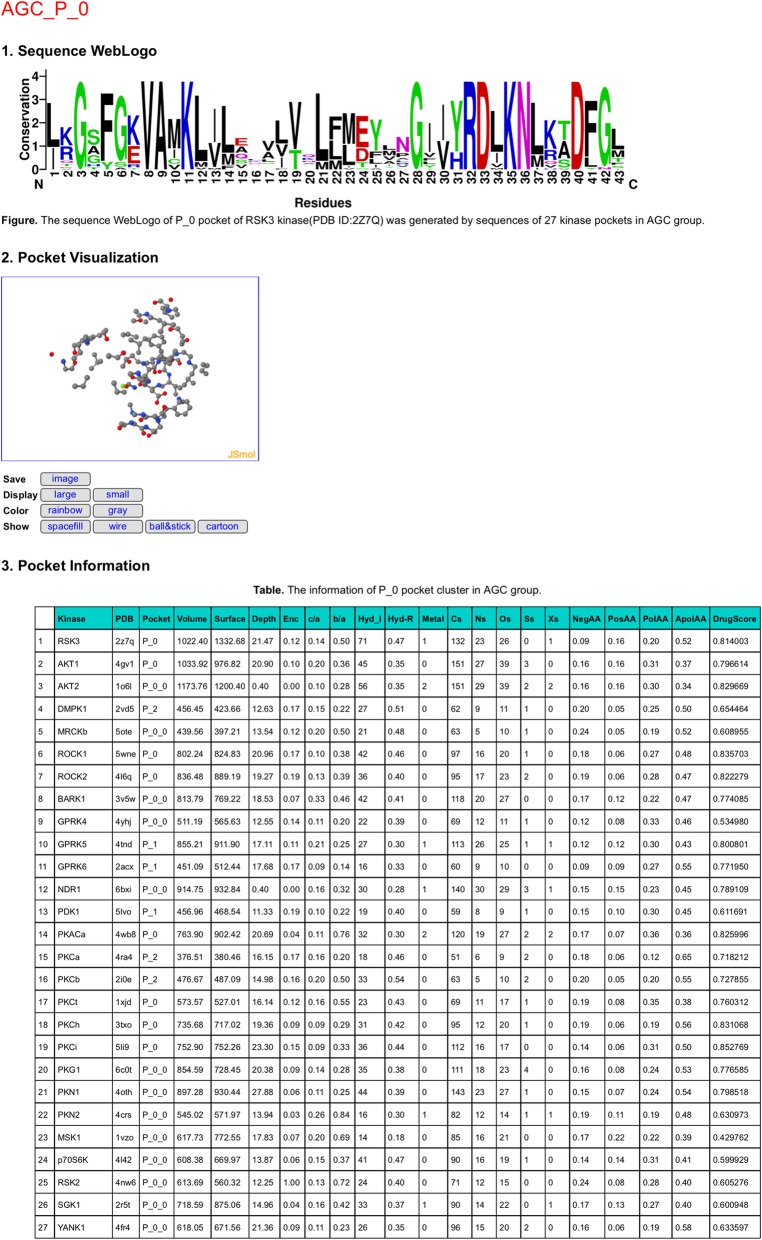
The pocket information of AGC_P_0. The information of AGC_P_0 pocket cluster contains three parts: Sequence WebLogo, Pocket Visualization, and Pocket Information. (1) A WebLogo plot was generated to show the sequence conservation of the pocket. The overall height of the residues in WebLogo indicates the frequency and conservation at the corresponding position. For AGC_P_0 pocket, the G3, V8, A9, K11, G28, R32, D33, K35, N36, and D40 residues are highly conserved. (2) Users can scale and rotate the AGC_P_0 pocket structure. HKPocket provides four representations: “spacefill”, “wire”, “ball&stick”, and “cartoon”. The key residues can be highlighted in different colors. Users can also generate and save the picture. (3) A pocket information table contains the structural shape (volume, surface, depth, etc.), sequence (negative amino acid ratio, polar amino acid ratio, etc.), atom (the number of metals, carbons, etc.), hydrophilic-hydrophobic, critical interactions and druggability information of cluster pockets

The Search module (Fig. 5) consists of two parts: one pulldown search box and a summary table of pocket clusters. In the pulldown search box, users can select the pocket cluster by group, catalytic/non-catalytic, and pocket cluster information. For example, Fig. 6 shows the detail information for AGC_P_0. (1) A WebLogo plot was generated to show the pocket sequence conservation. The overall height of the sequences in WebLogo indicates the frequency and conservation at the corresponding position. For AGC_P_0 pocket, the G3, V8, A9, K11, G28, R32, D33, K35, N36, and D40 residues are highly conserved. (2) Users can scale and rotate the pocket structures. HKPocket provides four representations: “spacefill”, “wire”, “ball&stick”, and “cartoon”. The key residues can be highlighted in different colors. Users can also generate and save the picture. (3) A pocket information table contains the structural shape (volume, surface, depth, etc.), sequence (negative amino acid ratio, polar amino acid ratio, etc.), atom (the number of metals, carbons, etc.), hydrophilic-hydrophobic, critical interactions, and druggability information.

The summary table contains the information of 91 pocket clusters including 8 catalytic pocket clusters and 83 non-catalytic pocket clusters. The identified pockets of a given kinase were sequentially numbered by P_0, P_1, P_2, etc. The pocket can be further divided into several small sub-pockets. For example, the pocket P_0 can be divided into two sub-pockets P_0_0 and P_0_1. Therefore, there are 11, 9, 13, 10, 14, 12, 8, 14 clusters of pockets in AGC, CAMK, CK1, CMGC, Other, STE, TK and TKL groups, respectively.

### Visualization module

In the visualization module, users can upload and investigate the pocket structure. The pocket structure will be visualized in four representations: “spacefill”, “wire”, “ball&stick”, and “cartoon”. The key residues can be highlighted in different colors. Users can scale and rotate the pocket structures. Users can also generate and save the picture.

### Download module

A summary table was provided in the download module. Users can download the individual group or all the pocket information. The download data consists four files: (1) The tables (xlsx format) with sequence and topology features for 91 pocket clusters; (2) The structure files (PDB format) with structure information of all pockets from 255 kinase proteins; (3) The pse files for structural detail visualization for 255 kinase proteins and their pockets. (4) The sequences files (txt format) of 255 kinase proteins in the HKPocket database.

### Links module

The Links module provides the other useful links of protein 3D structure resources, sequence alignment, molecular modeling, molecular dynamics, molecular dynamics, molecular visualization/analysis, and kinase-related database websites. These useful websites would be helpful to the kinase-related drug design.

### Tutorial module

The Tutorial module provides the introduction to use the HKPockt and the abbreviation for the HKPocket database.

### Contacts module

The Contacts module provides emails for users to comment or ask questions.

## Discussion

**Fig. 7:**
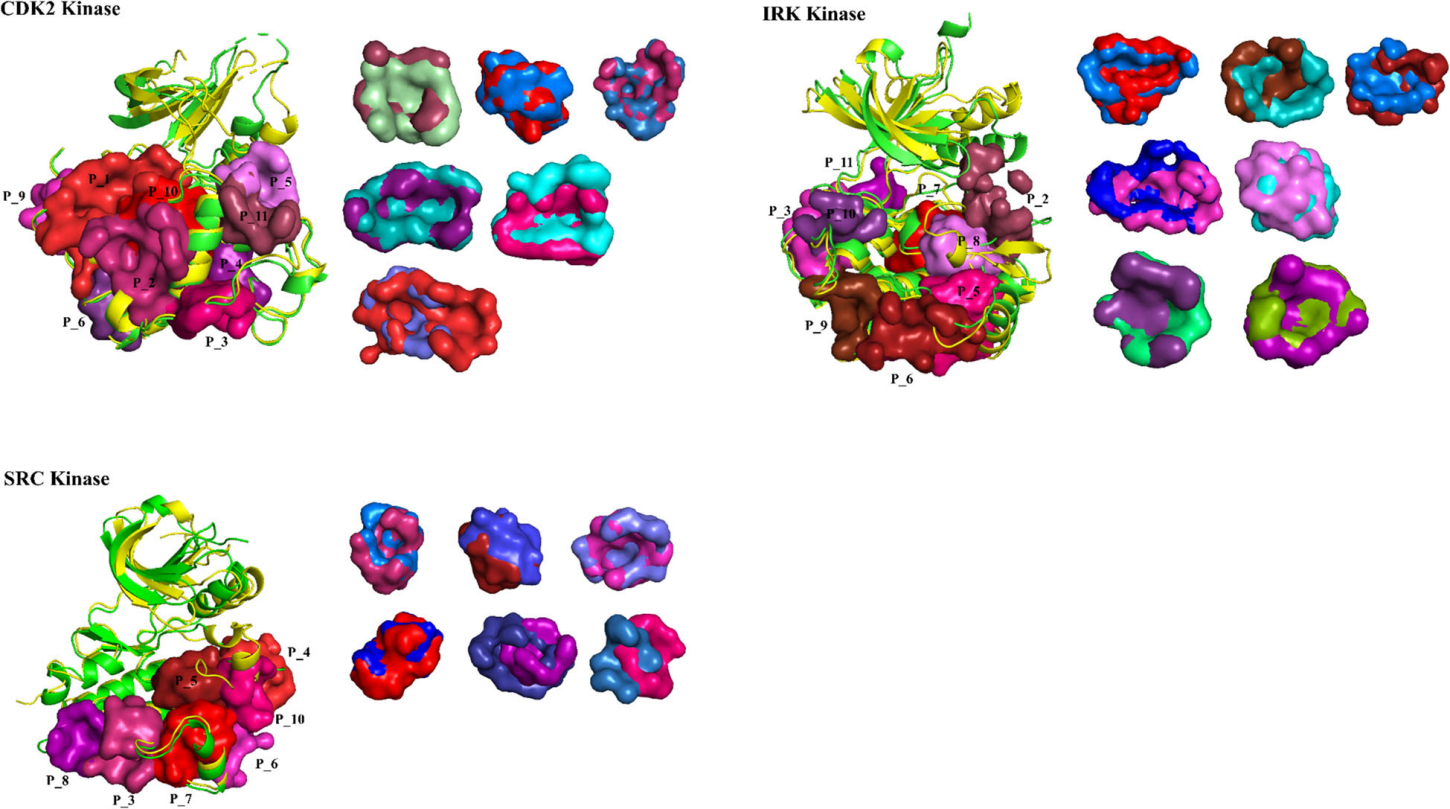
The structural analysis of non-catalytic pockets of CDK2, SRC, and IRK in active and inactive states. The active structure is colored in green. The inactive structure is colored in yellow. The results show 77% of non-catalytic pockets are very similar between active and inactive states

The kinase protein contains one N-terminal and one C-terminal lobe. The two lobes form the ATP-binding pocket. During the cell cycle, the kinase switches between the active (open) and inactive (closed) states due to the conformational transition of the DFG-loop. Previously, Kornev et al. analyzed the active and inactive of CDK2, SRC, and IRK structures [53, 54]. The results showed that there are some conformational changes in the catalytic region while fewer changes in the non-catalytic region. We analyzed the non-catalytic pockets of CDK2, SRC, and IRK kinase proteins in both active and inactive states. The CDK2, IRK, and SRC contain 9, 9, and 7 non-catalytic pockets in active states (Fig. 7). The results show 77% (6, 7 and 6) of non-catalytic pockets are very similar between active and inactive states. Therefore, the pocket information in HKPocket would be useful for allosteric drug design.

## Conclusions

The precision medicine initiative in kinase drug design is needed urgently due to the abnormal kinase activity could cause unexpected diseases. Müller et al. raised this question in 2015 and pointed out that the human kinome is now very well covered by the tertiary structure, making it possible to perform a comprehensive analysis of potential drug binding pockets for developing specific kinase drugs. In summary, we developed a well-analyzed human kinome pocket database with quantitative information of sequence, structure, interaction, and drug score. The HKPocket allows users to perform a systematic analysis of human kinase pockets for specific drug design. We hope the HKPocekt database will be useful for drug screen and optimization if the targeted pocket is known.

## Data Availability

The datasets generated and analyzed in the current study are available at http://zhaoserver.com.cn/HKPocket/HKPocket.html.

## Abbreviations

HKPocket: Human kinase pocket database
SVM: Support vector machine
